# M2 Macrophage-Derived Exosomes Promote Angiogenesis and Growth of Pancreatic Ductal Adenocarcinoma by Targeting E2F2

**DOI:** 10.1016/j.ymthe.2020.11.024

**Published:** 2020-11-20

**Authors:** Yuhan Yang, Zengya Guo, Weiwei Chen, Xiaofeng Wang, Meng Cao, Xuan Han, Kundong Zhang, Buwei Teng, Jun Cao, Weidong Wu, Peng Cao, Chen Huang, Zhengjun Qiu

**Affiliations:** 1 Department of General Surgery, Shanghai General Hospital, Shanghai Jiao Tong University School of Medicine, Shanghai 200080, People’s Republic of China; 2 Lianyungang Clinical College of Nanjing Medical University, The First People’s Hospital of Lianyungang, Lianyungang, Jiangsu, People’s Republic of China; 3 Affiliated Hospital of Integrated Traditional Chinese and Western Medicine, Nanjing University of Chinese Medicine, Nanjing, Jiangsu, China; 4 College of Pharmacy, Nanjing University of Chinese Medicine, Nanjing, Jiangsu, China

## Abstract

Pancreatic ductal adenocarcinoma (PDAC), one of the most aggressive tumors all over the world, has a generally poor prognosis, and its progression is positively correlated with the density of blood vessels. Recently, tumor-associated macrophages (TAMs) were proven to be beneficial for angiogenesis, but their mechanism of action remains unclear. Our study indicated that M2 macrophages were positively correlated with the microvessel density (MVD) of PDAC tissues, and M2 macrophage-derived exosomes (MDEs) could promote the angiogenesis of mouse aortic endothelial cells (MAECs) *in vitro*. At the same time, the M2 MDEs could also promote the growth of subcutaneous tumors and increase the vascular density of mice. Moreover, we also found that miR-155-5p and miR-221-5p levels in the M2 MDEs were higher than those in M0 MDEs, and they could be transferred into MAECs, as demonstrated by RNA sequencing (RNA-seq) and qPCR analysis. Our data confirmed the interaction between TAMs and the angiogenesis of PDAC by exosomes. Additionally, targeting the exosomal miRNAs derived from TAMs might provide diagnostic and therapeutic strategies for PDAC.

## Graphical Abstract

**Figure:**
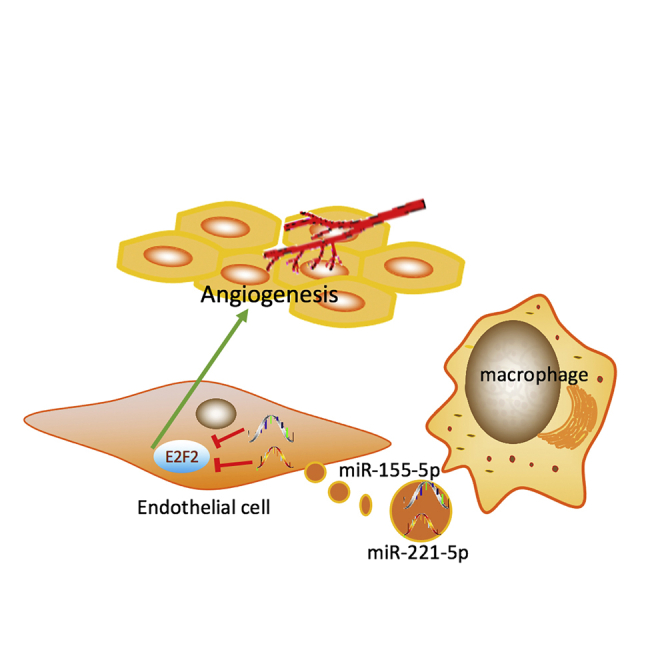

## Teaser

In this study, Qiu and colleagues found that M2 macrophage-derived exosomes could promote angiogenesis in pancreatic cancer, which revealed a new mechanism of communication between macrophages and vascular endothelial cells in tumors. This study provides a potential therapeutic target for pancreatic cancer.

## Introduction

Pancreatic cancer (PC), one of the most devastating malignancies, ranks fourth among all reasons to cause cancer death in the US.^1^ Angiogenesis, the process by which new capillaries grow from the pre-existing blood vessels, is associated with the growth and metastasis of numerous solid tumors, including PC.^2^ In general, PC is thought to be vascularized, and many studies have confirmed the positive correlation of microvessel density (MVD) with the progression of PC.^3^

Tumor-associated macrophages (TAMs) account for roughly 15%–20% of the total cellular tumor mass,^4^ and they have always been thought to have M2-like polarization and to be activated by T helper 2 (Th2) cytokines. In our recent study, hypoxic PC cell-derived exosomes were proven to promote M2 macrophage polarization.^5^ A previous study indicated that TAMs could induce the proliferation of endothelial cells and the formation of a vascular network in a vascular endothelial growth factor (VEGF)-dependent manner, and TAMs expressed VEGF-A in the perivascular area at the front of tumor invasion,^6^ which was confirmed to be helpful to increase MVD and hematogenous metastasis of tumors.^7^

Exosomes, the lipid bilayer membrane vesicles derived from the luminal membrane of multi-vesicular bodies, were proven to be beneficial for the communication between cells.^8^, ^9^, ^10^, ^11^ Typical exosomes often have a wide range of functional mRNAs, microRNAs (miRNAs), and proteins, and they play a key role in intercellular communication via transferring their genetic contents.^12^^,^^13^ Previous studies indicated that macrophage-derived exosomes (MDEs) significantly affected the proliferation, metastasis, and immune escape of tumors.^14^^,^^15^ Also, M2 MDEs might induce gemcitabine resistance in PC through delivering miR-365.^16^

In the present study, we demonstrated a mechanism of angiogenesis in PC, which was mediated by the shuttling of miRNAs between TAMs and endothelial cells through exosomes.

## Results

### M2 Macrophages Increase the Density of Microvessels in Tumor Tissues from Pancreatic Ductal Adenocarcinoma (PDAC) Patients

**Figure 1:**
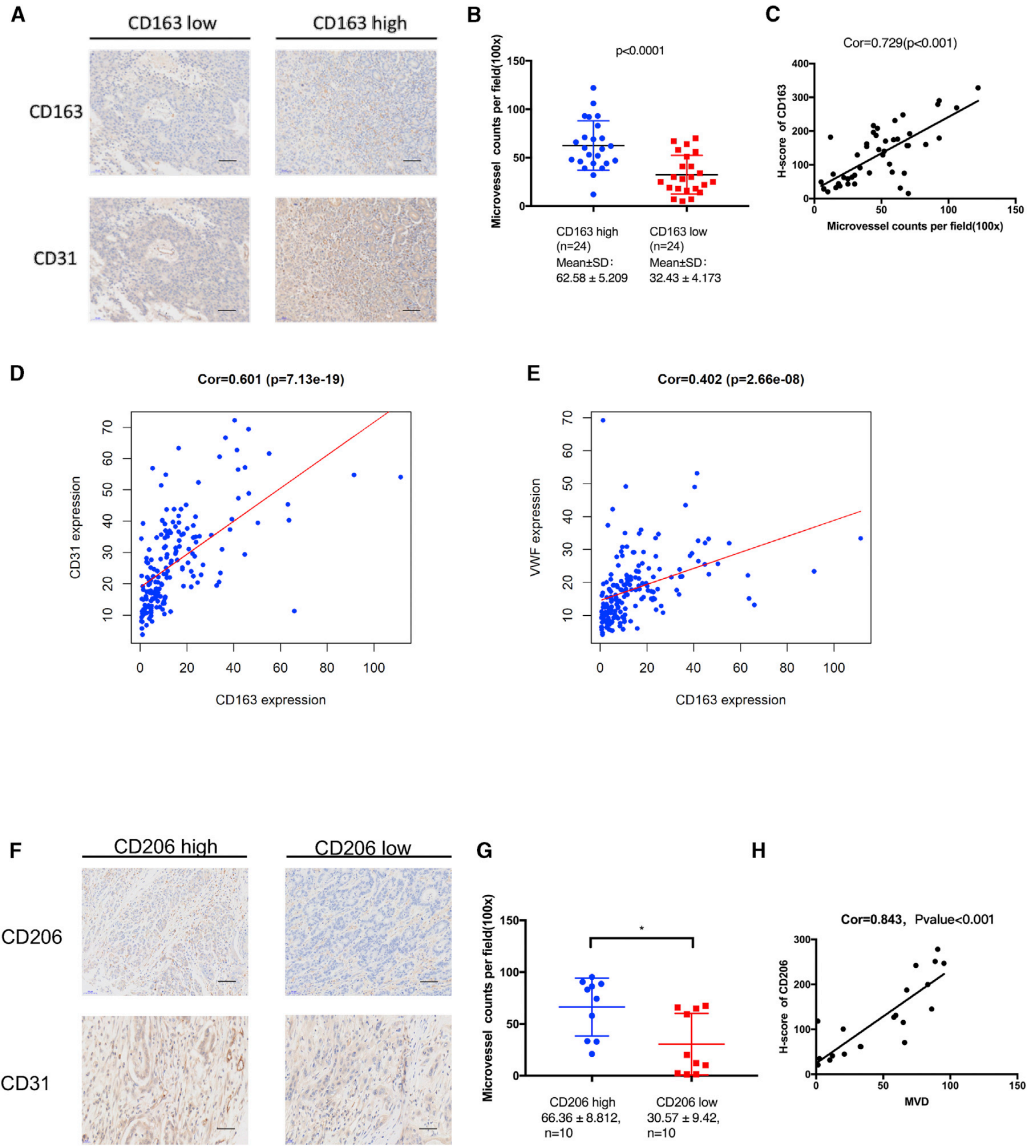
M2 macrophages Increase Microvessel Density in Tumor Tissues From PDAC Patients (A) Representative images of immunohistochemistry (IHC) staining for CD163 and CD31 in human PDAC tissues. Scale bars, 100 μm. (B) Quantification of microvessel counts per field. (C) Pearson correlation analysis of the H-score of CD163 and MVD in human PDAC tissues. (D and E) Pearson correlation analysis of the mRNA expression profiles of CD163 and CD31 (D, left), as well as CD163 and vWF (E, right) in 178 PDAC patients from TCGA. (F) Representative images of IHC staining for CD206 and CD31 in tumor tissues of nude mice. Scale bars, 100 μm. (G) Quantification of microvessel counts per field. (H) Pearson correlation analysis of the H-score of CD163 and MVD in tumor tissues of nude mice. ∗p < 0.05.

In this trial, CD31 and CD163 antibodies were first used to stain vascular endothelial cells and M2 macrophages, and then MVD and H-score were calculated. The results demonstrated that the MVD in PDAC tissues with a high level of CD163 was markedly enhanced compared to that in the PDAC tissues with a low level of CD163 (p < 0.001; Figures 1A and 1B). Additionally, there is a positive correlation between CD31 and CD163 levels (correlation [Cor] = 0.729, p < 0.001; Figure 1C).

The correlation between MVD (von Willebrand factor [vWF] and CD31 mRNA levels) and the CD163 mRNA level in 178 human PDAC patients obtained from The Cancer Genome Atlas (TCGA) was further determined. As shown in Figures 1D and 1E, significant correlations between the mRNA levels of CD163 and vWF (Cor = 0.402, p < 0.001), as well as CD163 and CD31 (Cor = 0.601, p < 0.001), were observed.

We subcutaneously injected Pan02 cells into nude mice and examined the CD206 and CD31 levels in tumor tissues. As shown in Figures 1F-H, the result showed that there were significant correlations between the levels of CD31 and CD206 (Cor = 0.843, p < 0.001).

Taken together, these results suggested that M2 macrophages are related to angiogenesis in PC.

### M2 MDEs Promote Angiogenesis *In Vitro*

**Figure 2:**
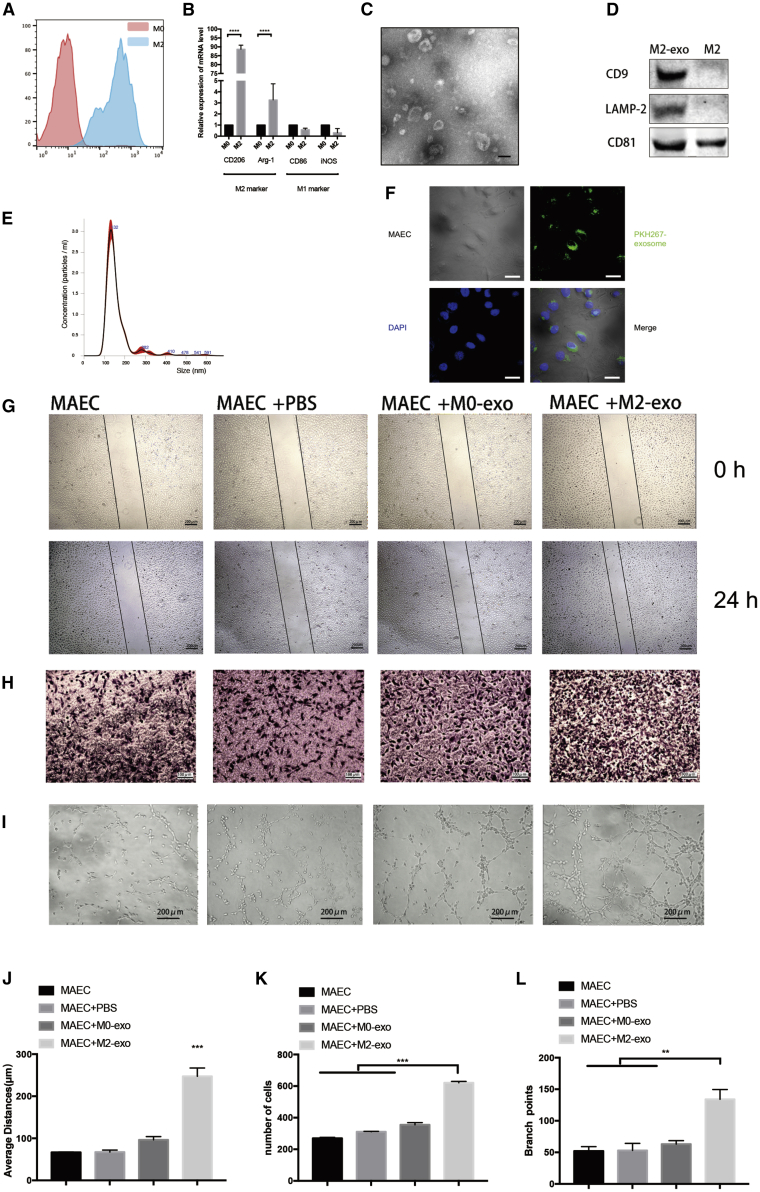
M2 Macrophage-Derived Exosomes Promote Angiogenesis *In Vitro* (A) Flow cytometry was used to detect the expression of the M2 macrophage marker CD206 in BMDMs. (B) qPCR was used to detect the mRNA level of M2 and M1 macrophage markers. (C) Electron microscopy images of exosomes isolated from conditioned medium of BMDMs. (D) Western blot of macrophage-derived exosomes. (E) Nanoparticle tracking analysis (NTA) of macrophage-derived exosomes. (F) Immunofluorescence images show that MAECs internalized PKH267-labeled macrophage-derived exosomes. (G) Representative micrographs of the transwell assay (original magnification, ×100). The numbers of cells were calculated per high-power field from three independent experiments. (H) Representative micrographs of tube formation assay (original magnification, ×200). The numbers of branch points were calculated by ImageJ. (I) Representative micrographs of the 24-h average distance of the wound-healing assay. (J–L) Data on transwell, wound-healing, and tube formation assays. ∗∗p < 0.01, ∗∗∗p < 0.001.

Our previous study found that the PC cell-derived exosomes could mediate M2 macrophage polarization,^5^ which prompted us to further explore whether M2 macrophages can induce angiogenesis in PDAC by exosomes. For TAMs, supernatants of Pan02 cells were added to macrophages. Then, qPCR and flow cytometry analyses demonstrated that the macrophages were positive for M2 markers, including Arg-1 and CD206. (Figures 2A and 2B).

Next, the media were purified through differential centrifugations and examined using transmission electron microscopy (TEM). To confirm that the source of the exosomes is from TAMs and not Pan02 cells, we collected the PBS for washing cell culture dishes before collecting exosomes and purified exosomes by ultracentrifugation. 100 μL of PBS was used to resuspend the exosomes. Western blot and electron microscopy indicated no exosomes remained after three washes (Figures S2C and S2D). TEM imaging showed that there were nanovesicles of various sizes (Figure 2C; Figure S2A), and their mean diameter was 132 nm at room temperature (Figure 2D).

Immunoblotting for the lysates from purified nanovesicles was performed using known exosomal markers.^17^ The results showed that CD9, CD63, and LAMP2 were expressed in these nanovesicles (Figure 2E; Figure S2B), suggesting that they were MDEs. Next, fluorescence microscopy was used to further confirm that endothelial cells could take up MDEs (Figure 2F).

Previous studies showed that M2 macrophages could facilitate the formation of blood vessels through VEGF-A and other cytokines. We incubated the exosome-free supernatant of M2 macrophages with endothelial cells and found that the effects of M2 macrophages on angiogenesis were weakened after the removal of exosomes (Figures S2E–S2J).

Migration, wound-healing, and tube formation assays were performed to examine the angiogenic ability of mouse aortic endothelial cells (MAECs), thereby investigating the effect of MDEs on vessel endothelial cells. As shown in Figures 2G–2L, MAECs co-cultured with M2 MDEs significantly increased the angiogenic ability of MAECs compared to co-cultures with M0 MDEs. We filtered the conditioned medium (CM) of the cancer cells before running it on TAMs by ultracentrifugation (110,000 × *g*). Tube formation, migration, and wound-healing assays indicated that the effect of MDEs was no different between the filtered and non-filtered groups (Figures S2K–S2P), which confirmed the effect of MDEs on angiogenesis.

### M2 MDEs Promote the Growth and Angiogenesis of Tumors *In Vivo*

**Figure 3:**
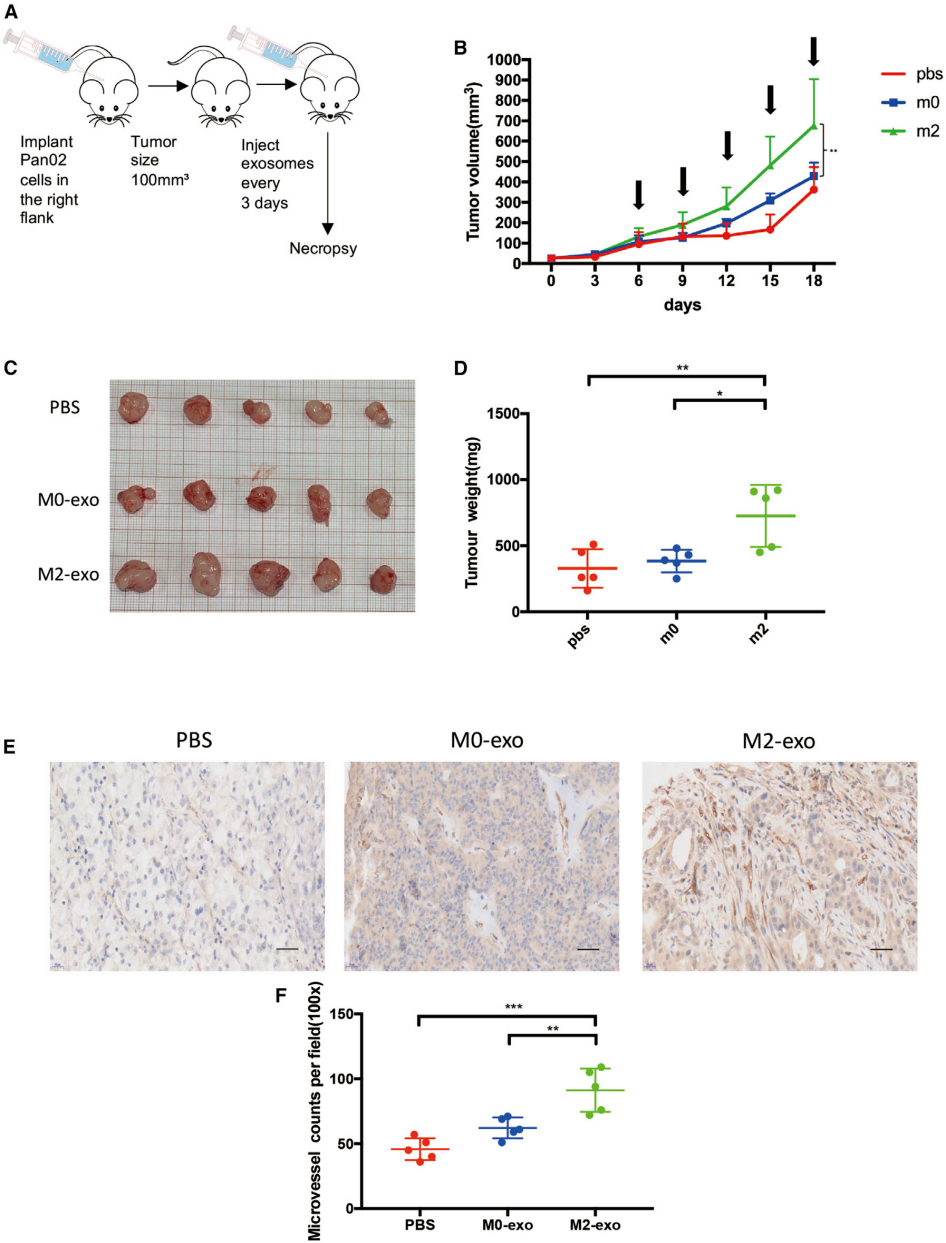
M2 Macrophage-Derived Exosomes Promotes Tumor Growth and Angiogenesis *In Vivo* (A) Model diagram of mouse tumorigenesis model. (B) The tumor growth curve shows the tumor size measured every 3 days, and the arrows represent the injected exosomes. (C) Tumor image of each group. (D) The weight of tumors in each group. (E) Representative IHC graph of tumor tissue in indicated groups. Scale bars, 100 μm. (F) MVD in each group of tumor tissue. ∗p < 0.05, ∗∗p < 0.01, ∗∗∗p < 0.001.

To investigate whether MDEs induce PDAC angiogenesis, Pan02 and 266-6 cells were subcutaneously injected into nude mice, and then M2 MDEs and M0 MDEs were injected into tumor centers every 3 days as shown in Figure 3A. To determine the bioavailability of macrophage exosomes in tumors, we synthesized a unique 75-nt-long double-stranded DNE (dsDNA) “barcode fragment,” which was transfected to macrophages. Then, barcodes in MDEs (Figure S3K) and endothelial cells (Figure S3L) of tumors were quantified by qPCR. Figure 3B shows the growth kinetics of tumors in each group. As shown in Figures 3C and 3D, tumor weights were much heavier in the M2 MDE group than those in the M0 MDE and control groups. Next, CD31 antibody was used to stain the vascular endothelial cells in tumors, and then MVD was calculated. The results showed that the MVD in the tumor tissues treated with M2 MDEs was markedly enhanced compared to that in the tumor tissues treated with M0 MDEs or PBS (Figures 3E and 3F). We also used the 266-6 cell line to a construct tumor-bearing mouse model (Figures S3A–S3E). These results collectively suggested that M2 MDEs could promote tumor growth and angiogenesis *in vivo*.

### MDEs Transport miR-155-5p and miR-221-5p into Endothelial Cells and Promote Angiogenesis

The evidence indicated that exosomes were enriched in miRNAs. To investigate which miRNAs were transferred into endothelial cells, we analyzed the miRNA contents in M0 and M2 macrophages using Illumina HiSeq 2500, and we found that miR-146, miR-155, miR-221, miR-320, and miR-382 were reported to promote angiogenesis^18^^,^^19^ and they were markedly upregulated in M2 relative to M0 macrophages (Figure S4).

**Figure 4:**
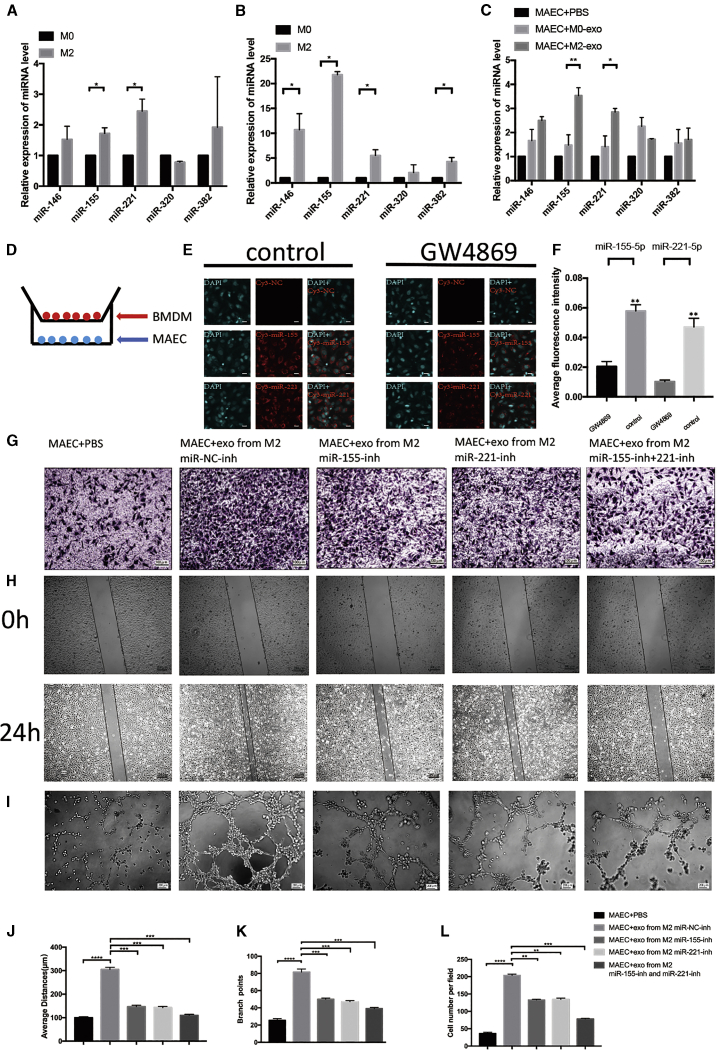
MDEs Transport miR-155-5p and miR-221-5p into Endothelial Cells and Promote Angiogenesis (A) qPCR indicated the relative miRNA level in M0 and M2 macrophages. (B) Relative level of miRNA in M0 and M2 macrophage-derived exosomes. (C) Relative level of miRNA in MAECs treated with exosomes for 24 h. (D) Schematic illustration of the *in vitro* co-culture system. (E) Immunofluorescence images show the Cy3-miRNA in MAECs of each group. Scale bars, 30 μm. (F) Average fluorescence intensity of exosomes. (G) Representative micrographs of the transwell assay (original magnification, ×100). The numbers of cells were calculated per high-power field from three independent experiments. (H) Representative micrographs of the 24-h average distance of the wound-healing assay. (I) Representative micrographs of the tube formation assay (original magnification, ×200). The numbers of branch points were calculated by ImageJ. (J–L) Data on transwell, wound-healing, and tube formation assays. ∗p < 0.05, ∗∗p < 0.01, ∗∗∗p < 0.001, ∗∗∗∗p < 0.0001.

The relative abundances of miR-146, miR-155, miR-221, miR-320, and miR-382 in M2 and M0 macrophages as well as their exosomes were compared using qPCR. The results showed that M2 macrophages were enriched in miR-155 and miR-221, while M2 MDEs were enriched in miR-146, miR-155, miR-221, and miR-382 (Figures 4A and 4B).

We co-cultured MAECs with M2 MDEs or M0 MDEs for 24 h and detected the relative miRNA levels by qRT-PCR. As Figure 4D demonstrates, miR-155 and miR-221 were most differently expressed in the MAECs co-cultured with M2 MDEs relative to M0 MDEs or PBS (Figure 4C).

Next, the Cy3-labeled miR-155 and miR-221 were transfected into the M2 macrophages treated with GW4869 (a vesicular inhibitor) for 24 h. Then, MAECs were co-cultured with macrophages using a 0.4-μm pore size transwell. Following 48 h of co-culture, Cy3-positive MAECs were detected using immunofluorescence. The results showed that the fluorescence intensity in the control group was much higher than that in GW4869 group, suggesting that miR-155 and miR-221 were transferred from M2 macrophages into endothelial cells via exosomes (Figures 4D–4F).

We next transfected miR-155-5p and/or miR-221-5p inhibitors into macrophages and collected their exosomes. Anti-miRNA efficiency was determined using a luciferase assay (Figures S5A and S5B). The above results indicated that the inhibition of miR-155-5p and miR-221-5p decreases the angiogenic ability of M2 MDEs (Figures 4G–4L). Additionally, we transfected miR-155-5p and/or miR-221-5p mimics into M0 macrophages and collected their exosomes. As shown in Figures S5C–S5H, overexpression of miR-155-5p or miR-221-5p in M0 MDEs can improve the angiogenic ability of M0 MDEs.

### Exosomal miR-155-5p and miR-221-5p Promote Angiogenesis in an E2F2-Dependent Manner

**Figure 5:**
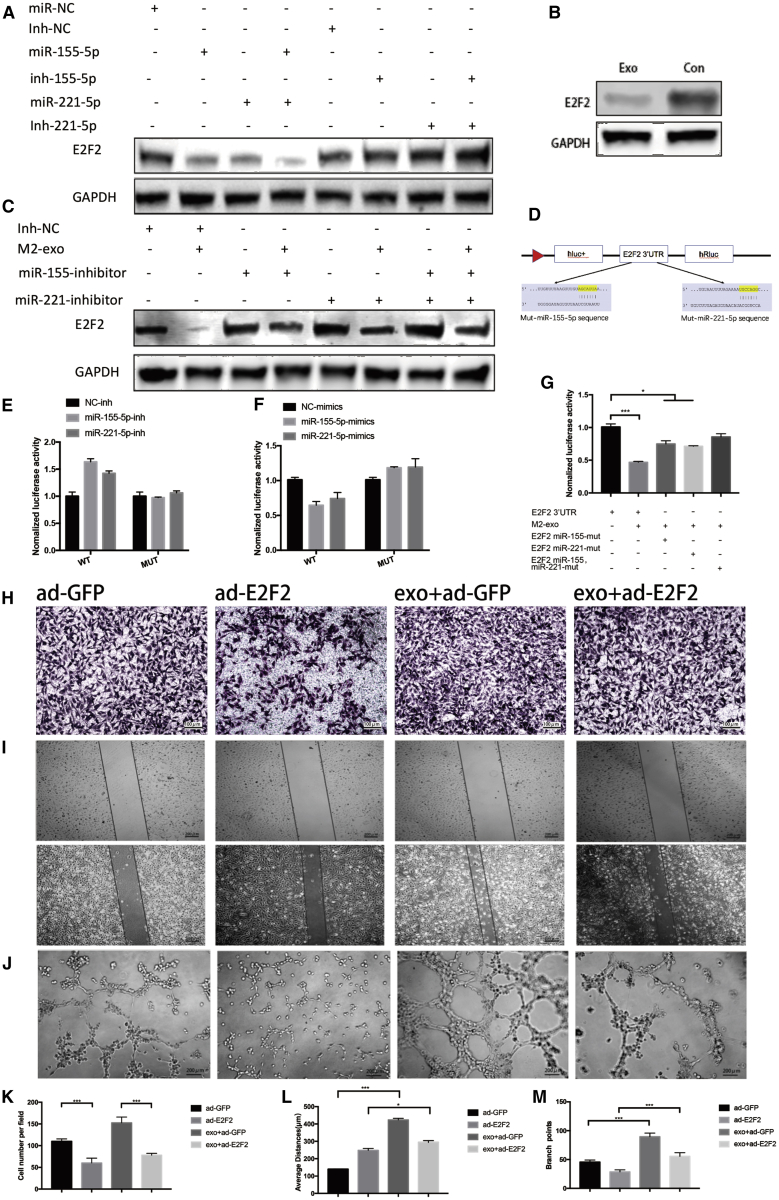
Exosomal miR-155-5p and miR-221-5p Promote Angiogenesis in an E2F2-Dependent Manner (A) Protein expression analysis of E2F2 in MAECs 72 h after transfection with miR-155-5p mimics, miR-221-5p mimics, or the negative control (mir-NC) and miR-155-5p inhibitor, miR-221-5p inhibitor, or the negative control (Inh-NC). (B) Protein levels of E2F2 after 72-h incubation of MAECs with exosome-free medium or M2-exo. (C) E2F2 protein levels of MAECs after incubation with M2-exo, miR-155-5p-inhibitor, and/or miR-221-5p-inhibitor. (D) In the 3′ UTR region of E2F2, the predicted binding region and mutation of mir-155-5p and mir-221-5p are shown. (E) miR-221-5p-inhibitor or miR-155-5p-inhibitor and plasmid containing the E2F2 3′ UTR region were co-transfected into MAECs, and their relative luciferase activities were detected. (F) miR-155-5p or miR-221-5p and plasmid containing the E2F2 3′ UTR region were co-transfected into MAECs, and their relative luciferase activities were detected. (G) Plasmids containing the wild-type E2F2 3′ UTR or its mutation at the predicted miR-155-5p and/or miR-221-5p target sequences were transfected into MAECs, along with M2 macrophage-derived exosomes, and the relative luciferase activities were detected. (H) Representative micrographs of the transwell assay (original magnification, ×100). The numbers of cells were calculated per high-power field from three independent experiments. (I) Representative micrographs of the tube formation assay (original magnification, ×200). The numbers of branch points were calculated by ImageJ. (J) Representative micrographs of the 24-h average distance of the wound-healing assay. (K–M) Data on transwell, wound-healing, and tube formation assays. ∗p < 0.05, ∗∗∗p < 0.001.

miRNAs have been shown to bind to the 3¢ UTR of genes, thereby regulating the expressions of mRNAs and proteins.^20^ In this trial, the database starBase 2.0 was used to predict the target for miR-155-5p and miR-221-5p. The results showed that there were a total of 27 common targets for miR-155-5p and miR-221-5p (Figure S5I), and among these 27 genes E2F2 was reported to inhibit endothelial cell angiogenesis.^21^ To further confirm the roles that miR-155-5p and miR-221-5p played in E2F2 expression, miR-155-5p and miR-221-5p inhibitors and their mimics were transfected into MAECs. The results demonstrated that miR-155-5p and miR-221-5p mimics significantly reduced E2F2 expression. In contrast, their inhibitors slightly increased E2F2 expression, while they did not affect the mRNA expression of E2F2 (Figure S5J). In addition, the co-transfection of miR-155-5p mimic and miR-221-5p mimic presented a stronger effect than when used alone (Figure 5A). Following exposure of MAECs to M2 MDEs, E2F2 expression markedly decreased (Figure 5B). In contrast, co-transfecting miR-221-5p and miR-155-5p inhibitors into MAECs could rescue the decreased E2F2 expression (Figure 5C). Next, wild-type or miRNA binding site mutant E2F2 3′ UTR-driven luciferase vector and miR-155-5p or miR-221-5p mimics were co-transfected into MAECs (Figure 5D) to investigate whether E2F2 is a common target for miR-155-5p and miR-221-5p. Compared to the control, the overexpression of miR-155-5p or miR-221-5p significantly suppressed the luciferase activity of wild-type E2F2 3′ UTR. Moreover, the suppression was reversed by miR-155-5p and miR-221-5p binding site mutations (Figures 5C and 5D). Conversely, the co-transfection of miR-155-5p inhibitor (Inh-miR-21-5p) or miR-221-5p inhibitor (Inh-miR-155-5p) markedly enhanced the Renilla luciferase activity of the reporter with wild-type 3′ UTR of E2F2, while it did not affect that of the mutant reporter (Figures 5E and 5F). On the contrary, M2 MDEs significantly reduced the Renilla luciferase activity of the reporter with wild-type 3′ UTR of E2F2, while they did not affect that of the reporter containing both miR-155-5p and miR-221-5p binding sequence mutants (Figure 5G). Additionally, we treated MAECs with M0-derived exosomes and with miR-155-5p and miR-221-5p and detected the E2F2 level with western blot in MAECs. As shown in Figure S5K, M1-derived exosomes plus miR-155-5p and miR-221-5p could decrease the level of E2F2 of MAECs, but at a higher level than M2-exo-treated MAECs. To further confirm whether the decreased E2F2 by exosomes can contribute to increasing the angiogenic ability of MAECs, MAECs were infected using adenoviral vector E2F2-GFP or adenoviral vector GFP, after which transfection efficiency was determined using western blotting (Figure S5L). The results demonstrated that the overexpression of E2F2 significantly suppressed the increased angiogenic ability of MAECs treated with M2 MDEs (Figures 5H–5M).

Taken together, our findings suggested that E2F2 might be the target for miR-155-5p and miR-221-5p in M2 MDEs, which further induced angiogenesis in MAECs.

### M2 Macrophage-Derived Exosomal miR-155-5p and miR-221-5p Promote Angiogenesis and Growth of PDAC *In Vivo*

**Figure 6:**
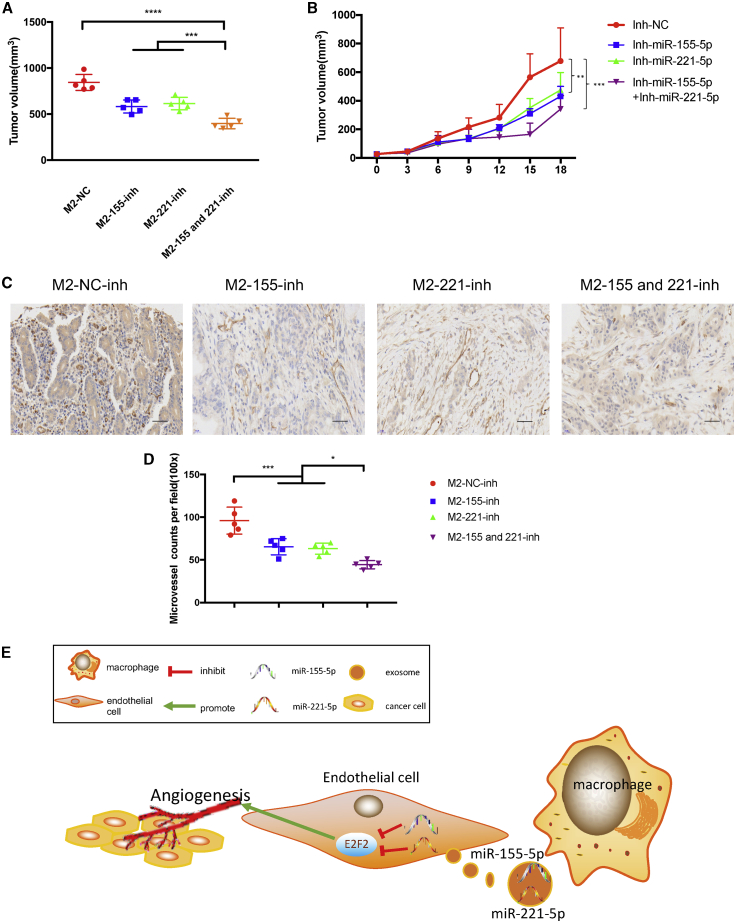
M2 Macrophage-Derived Exosomal miR-155-5p and miR-221-5p Promote Angiogenesis and Growth of PDAC *In Vivo* (A) Weight of tumors in each group. (B) The tumor growth curve shows the tumor size measured every 3 days. (C) Representative IHC graph staining for CD31 in tumor tissue of nude mice. Scale bars, 100 μm. (D) Dot plot shows the MVD in tumor tissue of nude mice by CD31. (E) Schematic model of M2 MDE promoting PDAC angiogenesis and growth by exosomal miR-155-5p and miR-221-5p by targeting E2F2. ∗p < 0.05, ∗∗∗p < 0.001, ∗∗∗∗p < 0.0001.

The results showed that the inhibitions for miR-155-5p and miR-221-5p disabled M2 MDEs, thereby increasing PDAC growth (Figures 6A and 6B). We also detected the CD31 level in tumor mice using immunohistochemistry assays and found that the inhibitions for miR-155-5p and miR-221-5p disabled M2 MDEs, thereby enhancing the MVD in PDAC (Figures 6C and 6D). Figures S3F–S3J show the above results for the 266-6 model. In summary, we have demonstrated that M2 macrophages had positive correlation with the MVD of human PC. M2 MDEs carried miR-155-5p and miR-221-5p to endothelial cells, which promoted the angiogenesis in PDAC by targeting E2F2 (Figure 6E).

## Discussion

In the microenvironment of tumors, macrophages play important roles. Many studies have shown that M2 macrophages can promote the progression, metastasis, and drug resistance of PC.^22^ Exosomes are lipid double-coated extracellular vesicles containing proteins and nucleic acids, especially miRNAs. They have been proven to mediate the material transfer and information communication between cells.^23^

Immature and distorted neovascularization is an important feature of solid tumors. The increases in vascular density and VEGF-A levels promote the development and metastasis of tumors. Although PC is often hypovascular due to large amounts of fibrotic stromata, many studies have shown a positive correlation between vascular density and the progression of PC. A study by Huang et al.^24^ showed that interleukin (IL)-35-mediated angiogenesis promoted the development of PC. Additionally, the success of anti-VEGF treatment in colorectal cancer and other tumors provided hope for the application of the anti-angiogenesis therapy for PC.^2^ However, the poor efficacy of traditional anti-VEGF drugs, such as sorafenib, in PC suggests that the mechanism of angiogenesis in PC needs to be explored.

Recent studies have shown that M2 macrophages can mediate drug resistance, invasion, metastasis, and immunosuppression of tumors by releasing exosomes. Specifically, Binenbaum et al.^16^ found that M2 MDEs could facilitate the resistance of PC cells to gemcitabine; Lan et al.,^15^ Zheng et al.,^25^ and Yin et al.^26^ found that M2 MDEs could facilitate the migration and invasion of cancer cells. Moreover, a recent study indicated that the modification of macrophage vesicles had the value of targeted therapy for cancers, in which molecularly engineered MDEs could target and alleviate inflammation in atherosclerotic lesions via a surface-binding chemokine receptor and anti-inflammatory cytokines at the same time.^27^

Many studies also showed that M2 macrophages could promote angiogenesis,^28^ and TAM infiltration was negatively correlated with the prognosis of the patients who received anti-angiogenic therapy.^29^ In our study, the correlation between M2 macrophages and angiogenesis was confirmed using 48 human PDAC tissues, subcutaneous tumor tissues of mice, and TCGA database. Thus, it is necessary to further explore whether the communication between M2 macrophages and vascular endothelial cells depends on exosomes in PC.

Compared with M0 MDEs, M2 MDEs can promote the formation of blood vessels *in vivo* and *in vitro* and promote the progress of tumors. Given that the traditional anti-VEGF drugs often had poor efficacy for PC, M2 MDEs with the function of promoting the angiogenesis of TAM exocrine vesicles might be a novel therapeutic target for PC.

miRNAs, small noncoding RNAs with about 22 nt, can regulate 3′ UTR binding to specific target gene mRNAs, thereby inhibiting gene expression translation or causing degradation. miR-155, one of the best conserved and multifunctional miRNAs, is primarily characterized by its overexpression in multiple diseases, such as malignant tumors.^30^, ^31^, ^32^, ^33^ At the same time, it can also mediate tumor invasion, metastasis, angiogenesis, and drug resistance. Moreover, the overexpression of miR-221 is thought to be negatively correlated with the prognosis of patients.^34^ Our study indicated that miR-155-5p and miR-221-5p expressions in exosome increased after M2 macrophage polarization, and they could be transferred into endothelial cells through exosomes. The inhibition for miR-155-5p and miR-221-5p in exosomes impaired M2 MDE-promoted angiogenesis, which was confirmed *in vitro* and *in vivo*. The above results suggested that M2 macrophage-derived exosomal miR-155-5p and miR-221-5p played crucial roles in the interaction between macrophages and endothelial cells, thereby promoting PDAC progression.

The E2F family of transcription factors were initially found to play key roles in cell cycle control by activating or inhibiting a group of response genes. Among the E2F family, the inhibition of E2F2 expression was proven to induce angiogenesis in cardiovascular disease.^33^ Moreover, the endothelial proliferation markedly increased in mice after E2F2 knockout.^21^ Our study showed that the inhibition for E2F2 M2 macrophage-derived exosomal miR-155-5p and miR-221-5p promoted angiogenesis, leading to PDAC progression *in vivo*. Our results also indicated that M2 MDEs suppressed E2F2 expression in endothelial cells, thereby promoting angiogenesis. As mentioned above, miRNAs could bind to the 3′ UTR of genes, thereby inhibiting gene expressions and then degrading mRNAs. Our previous study has shown that miR-155-5p could affect the invasiveness and migration of PC cells via regulating the STAT3 signal mediated by SOCS1.^35^ In our study, the E2F2 level in tumors injected with M2 MDEs was also higher than that in the tumors injected with M0 MDEs or PBS. The matrix caused by a large number of fiber deposition often makes chemotherapy drugs and anti-angiogenic drugs have poor efficacies for PC. One of the implications of our study is to provide the possible strategy of anti-angiogenesis therapy for PDAC by the immune transfer of antagonists miR-155-5p and miR-221-5p into primary tumors via macrophages.

In summary, we have demonstrated that M2 macrophages had a positive correlation with the MVD of human PC. M2 MDEs were more enriched in miR-155-5p and miR-221-5p compared to M0 MDEs, which promoted the angiogenic ability of endothelial cells. Our data revealed the action mechanism of TAMs in the angiogenic progression of PDAC and highlighted the mechanism as a diagnostic and therapeutic target for the anti-angiogenesis of PDAC.

## Materials and Methods

### Cell Lines and Cell Culture

Pan02 and 266-6 cells were purchased from the American Type Culture Collection (ATCC, Manassas, VA, USA). These cell lines have been identified by short tandem repeat (STR) DNA. The mycoplasma test was also negative. 0.10% fetal calf serum (FCS) with RPMI 1640 medium containing 1% penicillin and streptomycin was used to culture cell lines in a 5% carbon dioxide cell incubator.

For bone-marrow derived macrophages (BMDMs), mouse bone marrow was collected by flushing the femurs of C57BL/6 mice (8–10 weeks old) with cold PBS. After collection, red blood cells (RBCs) were lysed with RBC lysis buffer (Thermo Fisher Scientific, USA), and the remaining cells were washed twice with PBS. For induction of macrophage differentiation, sorted monocytes or bone marrow cells were cultured in RPMI 1640 or DMEM supplemented with 10% FBS and 20 ng/mL human or mouse macrophage colony-stimulating factor (M-CSF) (R&D Systems, USA). On day 6, the medium was changed to DMEM with 10% FBS. For TAMs, a supernatant of Pan02 cells was added for 2 days. M0 polarized macrophages were cultured in 10% DMEM. The MAECs were isolated and cultured as previously described.^36^

### Exosome Collection, Isolation, and Purification

When the macrophages were activated by the supernatant, Pan02 cells were added for 2 days. Next, we removed the previous medium and washed the dish with PBS three times to remove the residual exosomes. 10% exosome-free FBS DMEM was used to collect exosomes.

The exosomes in the medium were collected by ultracentrifugation.^37^ In short, first, 300 × *g* for 5 min, 2,000 × *g* for 5 min, and 12,000 × *g* for 30 min were used to remove the cell fragments and large vesicles in the supernatant, which were filtered with a 0.22-μm sieve. After that, the exosomes were collected by 110,000 × *g* ultracentrifugation, washed by PBS suspension, and then collected by 110,000 × *g*. Then, the exosomes were suspended in 200 μL of PBS suspension. The exosomes were also isolated by a precipitation method using ExoJuice (ExonanoRNA, Foshan, People’s Republic of China) according to the manufacturer’s instruction. Briefly, cell culture medium was harvested by centrifugation at 12,000 × *g* for 30 min. Then, the supernatant was placed in an ultracentrifuge tube and 1 mL of ExoJuice was added to the bottom of the centrifuge tube. After centrifuging at 100,000 × *g* for 70 min, the first 500 μL of liquid from the bottom of the tube was carefully collected and discarded. Then, the next 300 μL of liquid from the bottom was carefully collected and retained, which contained the purified exosomes.

### Exosome Identification by Nanoparticle Tracking Analysis (NTA), Electron Microscopy

By using a NanoSight analysis system (NanoSight, Navato, CA, USA), we determined the size and concentration of exosomes. Additionally, exosomes were stained with 2% glutaraldehyde, then dried on a copper net and imaged under an electron microscope.

### Exosome Internalization Experiments

After incubating the PKH267-labeled exosomes with MAECs for 24 h, the cells were fixed with 4% paraformaldehyde. Then, DAPI was used to label the nucleus and a microscope (Olympus, Japan) was used to detect PKH267 exosomes.

### Luciferase Activity Assay

2 × 10^4^ MAECs were cultured in 96-well plates, and we transfected miR-155-5p, miR-221-5p mimics, or inhibitor using riboFECT CP reagent (Ribobio, People’s Republic of China). Then, wild-type or miRNA binding site mutant E2F2 3′ UTR-driven luciferase vector (100 ng/well) was transfected into MAECs by Lipofectamine 3000 (Invitrogen, USA). After 24 h, luciferase of each well was detected using a Dual-Glo luciferase assay system (Promega, USA) and Centro XS3 LB 960 (Berthold, Germany). Firefly luciferase activity was used for normalization.

### Cell Migration, Wound-Healing, and Tube Formation Assays

In the cell migration assay, serum-free DMEM was first incubated with the upper chamber for 2 h to rehydrate this chamber. DMEM with 10% FBS was added into the inferior cavity. MAECs were treated with exosomes (10 μg of exosomes was resuspended in 100 μL of PBS and added with 1 × 10^5^ MAECs) or transfected with miRNA mimics or plasmids for 24 h. Then, MAECs were collected and diluted with serum-free DMEM (2 × 10^5^ added with 100 μL) and added to the upper cavity. After incubation for 24 h, five random visual fields (×200) were counted under a light microscope. Each experiment was repeated three times.

For the wound-healing assay, MAECs were treated with exosomes (10 μg of exosomes was resuspended in 100 μL of PBS and added with 1 × 10^5^ MAECs) or transfected with miRNA mimics or plasmids for 24 h. MAECs were then were harvested and seeded in a six-well plate at a density of 5 × 10^5^ cells/well. Then, we twice used a sterile 200-μL pipette to make a scratch and remove floating cells by using PBS. Images of the scratches were taken using an inverted microscope at ×100 magnification at 0 and 24 h after scratching. The average distance of the healed wound area was measured by comparing 24 h and 0 h using an Olympus IX71 microscope (Olympus).

A Matrigel tube formation assay was performed as previously described.^38^ In brief, MAECs were treated with exosomes (10 μg of exosomes was resuspended in 100 μL of PBS and added with 1 × 10^5^ MAECs) or transfected with miRNA mimics or plasmids for 24 h. MAECs were then harvested and resuspended with serum-free DMEM and seeded into 96-well plates (20,000 cells/well) precoated with growth factor reduced basement membrane matrix (BD Biosciences). Then, the plate was incubated at 37°C for 6–8 h. The tube formation was visualized under an inverted microscope. Enclosed networks of tube structures from three randomly chosen fields were recorded under a light microscope.

### RNA Sequencing Using an Illumina HiSeq 2500 System

Exosomes were isolated from one TAM culture. Total RNAs in exosomes were extracted using a total exosome RNA and protein isolation kit (Invitrogen/Life Technologies, Austin, TX, USA). The amount and quality of small RNA in the total RNA were tested by a NanoDrop spectrophotometer. Small RNA library construction and sequencing were performed by Gene Denovo Biotechnology (Guangzhou, People’s Republic of China). Then, the cDNA library was sequenced on an Illumina HiSeq 2500 (Gene Denovo Biotechnology). Illumina software was used for raw data analysis.

### Patient Samples

Our study was conducted in accordance with US Common Rule, and archived pancreatic duct adenocarcinoma specimens (n = 48) were collected under a Human Research Ethics Committee protocol at Shanghai General Hospital (Shanghai, People’s Republic of China) with patients’ written formal consent. These patients have been followed over time.

### Animals Experiments

All animal experiments were approved by the Ethics Committee for Animal Research of Shanghai Jiaotong University School of Medicine (Shanghai, People’s Republic of China). As previously reported, PC cells (Pan02, 5 × 10^6^ cells/animal, 266-6, 5 × 10^6^ cells/animal) were transplanted subcutaneously into the right flank of 4-week-old male nude mice. Tumor size was measured every 3 days by a digital caliper. On day 6, when the tumor volume reached approximately 100 mm^2^, the mice were randomly divided into each group, and corresponding exosomes (10 μg) were intratumorally injected into the center of tumors of mice every 3 days. After five injections, the mice were sacrificed and primary tumors were removed and the weights were recorded. After that, the tumors were excised for immunohistochemical staining for CD31, CD163, and CD206. CD31 was always used as a marker of endothelial cells. The method of quantifying the blood vessels was described previously.^39^

### Statistical Analysis

GraphPad Prism 7.0 software was used to conduct statistical analyses. The Pearson r correlation was taken for correlation analysis of mRNA expression from TCGA, H-score of tumor tissue, and MVD. A Student’s t test and one-way ANOVA were used to calculate the p value. p values <0.05 were considered statistically significant.

Other methods are found in Supplemental Materials and Methods.

## Supplemental Information

Document S1. Figures S1–S5 and Supplemental Materials and Methods

Document S2. Article plus Supplemental Information
