# Supplementary material for: M2 Macrophage-Derived Exosomes Promote Angiogenesis and Growth of Pancreatic Ductal Adenocarcinoma by Targeting E2F2
Source: Mol Ther. 2020 Nov 20;29(3):1226–38. doi: 10.1016/j.ymthe.2020.11.024 (PMC7934635; doi:10.1016/j.ymthe.2020.11.024)
Supplement: Document S1. Figures S1–S5 and Supplemental Materials and Methods [file mmc1.pdf]

YMTHE, Volume *29*

Supplemental Information

M2 Macrophage-Derived Exosomes Promote Angiogenesis and Growth of Pancreatic Ductal Adenocarcinoma by Targeting E2F2

Yuhan Yang, Zengya Guo, Weiwei Chen, Xiaofeng Wang, Meng Cao, Xuan Han, Kundong Zhang, Buwei Teng, Jun Cao, Weidong Wu, Peng Cao, Chen Huang, and Zhengjun Qiu

Supplemental data 1：The sequence of primers for qPCR.

All the miRNA reverse primers are 3’Universal Reverse primer in Ribo miRNA detect kit.

CD206

forward: 5′–GGGTTGCTATCACTCTCTATGC-3′,

reverse: 5′–TTTCTTGTCTGTTGCCGTAGTT-3′;

Arg-1

forward: 5′- GGTTTTTGTTGTTGCGGTGTTC-3′,

revered:5′-CTGGGATACTGATGGTGGGATGT-3′,

iNOS

forward: 5′–AGGGACAAGCCTACCCCTC-3′,

reverse: 5′–CTCATCTCCCGTCAGTTGGT-3′;

CD86

forward: 5′–CCAGCAGGCCTGGCTTATCC-3′,

reverse: 5′–AGCAAAGCAGGGGACACCAC-3′;

E2F2

forward：5’-ACGCTATGACACGTCGCTGG-3’

reverse：5’-CCTTCAGCTCCTGCCCCAAC-3’

All the miRNA reverse primers are 3’Universal Reverse primer in Ribo miRNA detect kit.

Mmu-MiR-155-5p

forward: 5’-UUAAUGCUAAUCGUGAUAGGGGUU-3’

Mmu-MiR-382-5p

forward: 5’-GAAGUUGUUCGUGGUGGAUUCG-3’

Mmu-MiR-221-5p

forward: 5’-ACCUGGCAUACAAUGUAGAUUUCUGU-3’

Mmu-MiR-146

forward: 5’-UGAGAACUGAAUUCCAUGGGUU-3’

Mmu-MiR-320

forward: 5’-GCCUUCUCUUCCCGGUUCUUCC-3’

u6

forward: 5'-CGC TTC GGC AGC ACA TAT ACT A-3'

Supplemental data 2

A

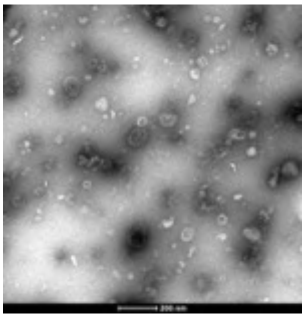

B

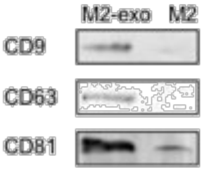

C

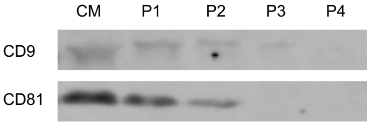

D

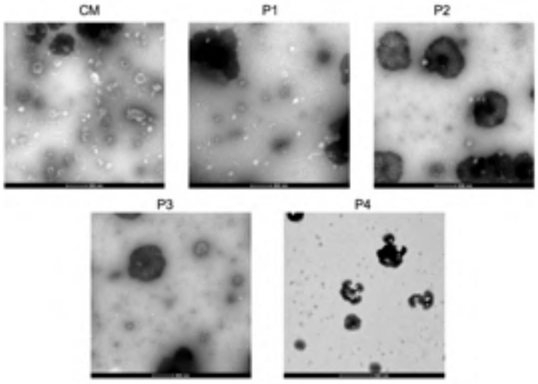

E M2-CM M2-CM-exo-free

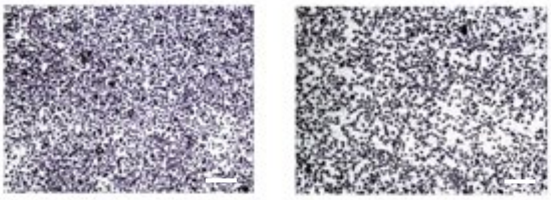

F

0h

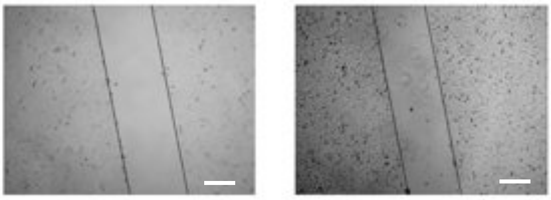

24h

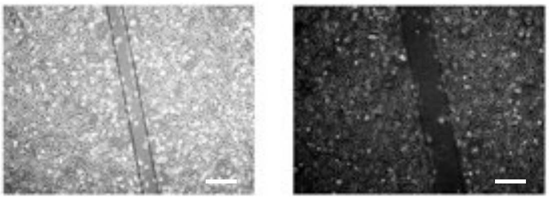

G

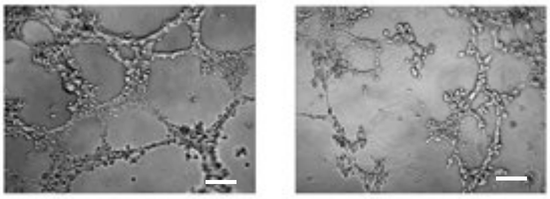

H

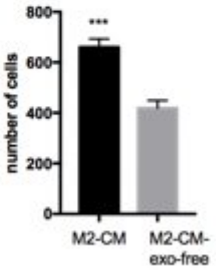

number of cells
800
600
400
200
0
***
M2-CM
M2-CM-exo-free

I

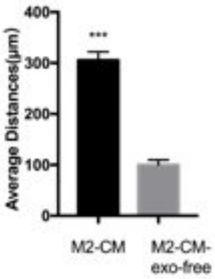

Average Distances(μm)
400
300
200
100
0
***
M2-CM
M2-CM-exo-free

J

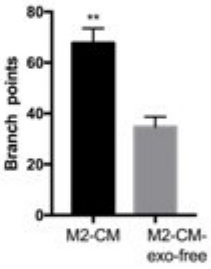

Branch points
80
60
40
20
0
**
M2-CM
M2-CM-exo-free

K

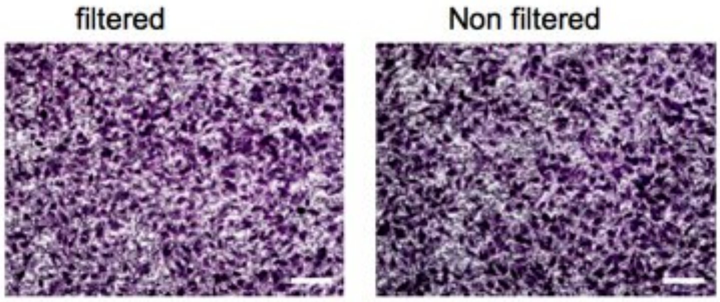

filtered
Non filtered

L

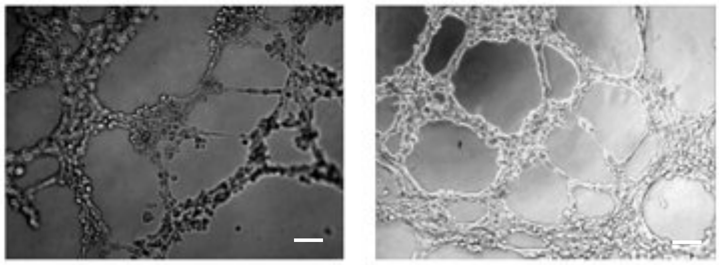

M

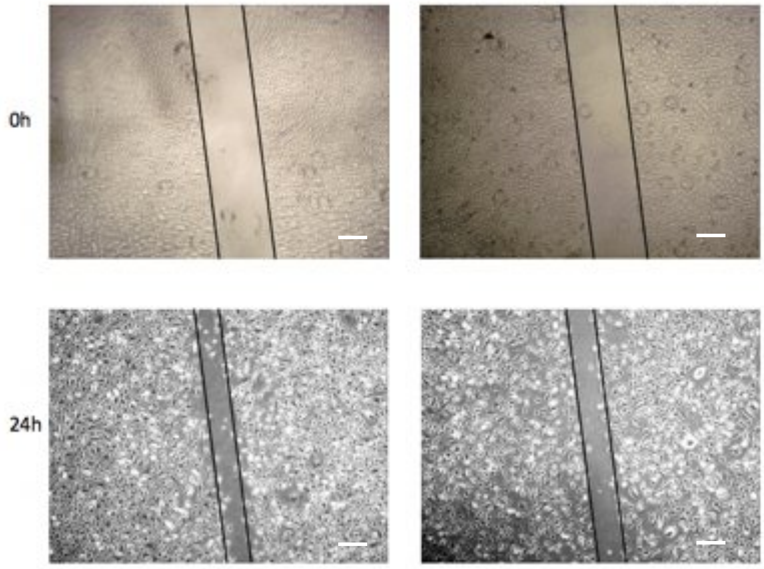

0h
24h

N

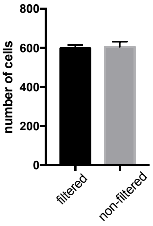

number of cells
800
600
400
200
0
filtered
non-filtered

O

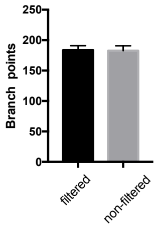

Branch points
250
200
150
100
50
0
filtered
non-filtered

P

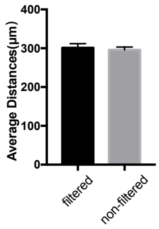

Average Distances(μm)
400
300
200
100
0
filtered
non-filtered

(a) Electron microscopy images of exosomes isolated from conditioned medium of BMDM by Exojuice. (b) Western-blot of macrophage-derived exosomes purified by Exojuice (c) Western blot of exosomes from pan02 CM and washing PBS.（P1-P4 represents PBS for the first to fourth washing）(d) Electron microscopy images of exosomes isolated from pan02 CM and PBS for washing dishes.（P1-P4 represents PBS for the first to fourth washing.）(e) Representative micrographs of the transwell assay (magnification 100×) (scale bar 200μm). Number of cells were calculated per high-power field from three independent experiments. (f) Representative micrographs of the 24h average distance of wound-healing assay (scale bar 100μm). (g) Representative micrographs of tube formation assay (magnification 200×) (scale bar 100μm). The number of branch points were calculated by imageJ. (h-j) Data statistics of transwell, wound-healing and tube formation assays. (k) Representative micrographs of the transwell assay (magnification 200×) (scale bar 100μm). Number of cells were calculated per high-power field from three independent experiments. (l) Representative micrographs of tube formation assay (magnification 200×) (scale bar 100μm). The number of branch points were calculated by imageJ. (m) Representative micrographs of the 24h average distance of wound-healing assay. (scale bar 100μm). (n-p) Data statistics of transwell, wound-healing and tube formation assays. **$p<0.01$, ***$p<0.001$

Supplemental data 3: M2 derived exosomal miR-155-5p and miR-221-5p induced angiogenesis and growth of pancreatic cancer in 266-6 in *vivo* model.

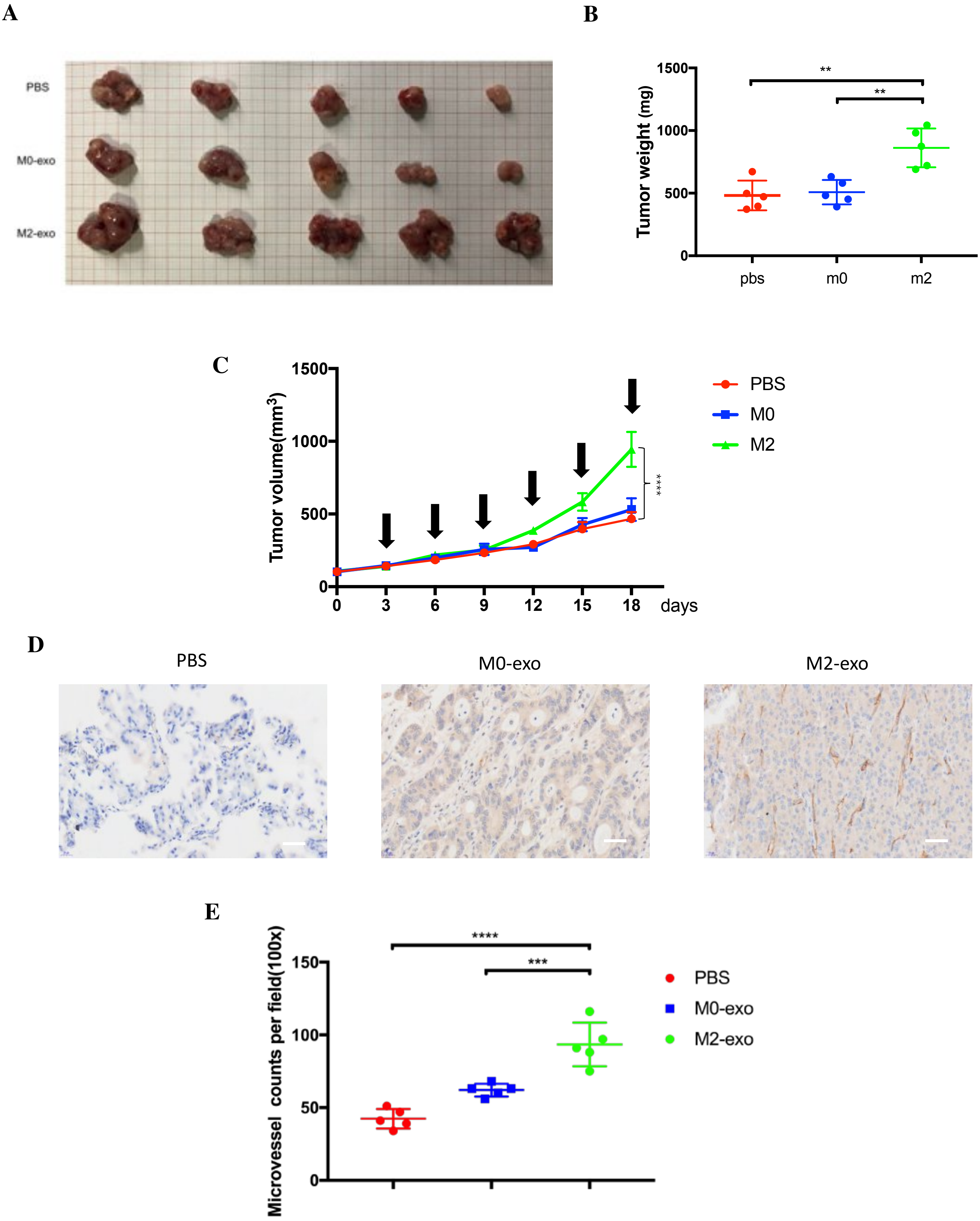

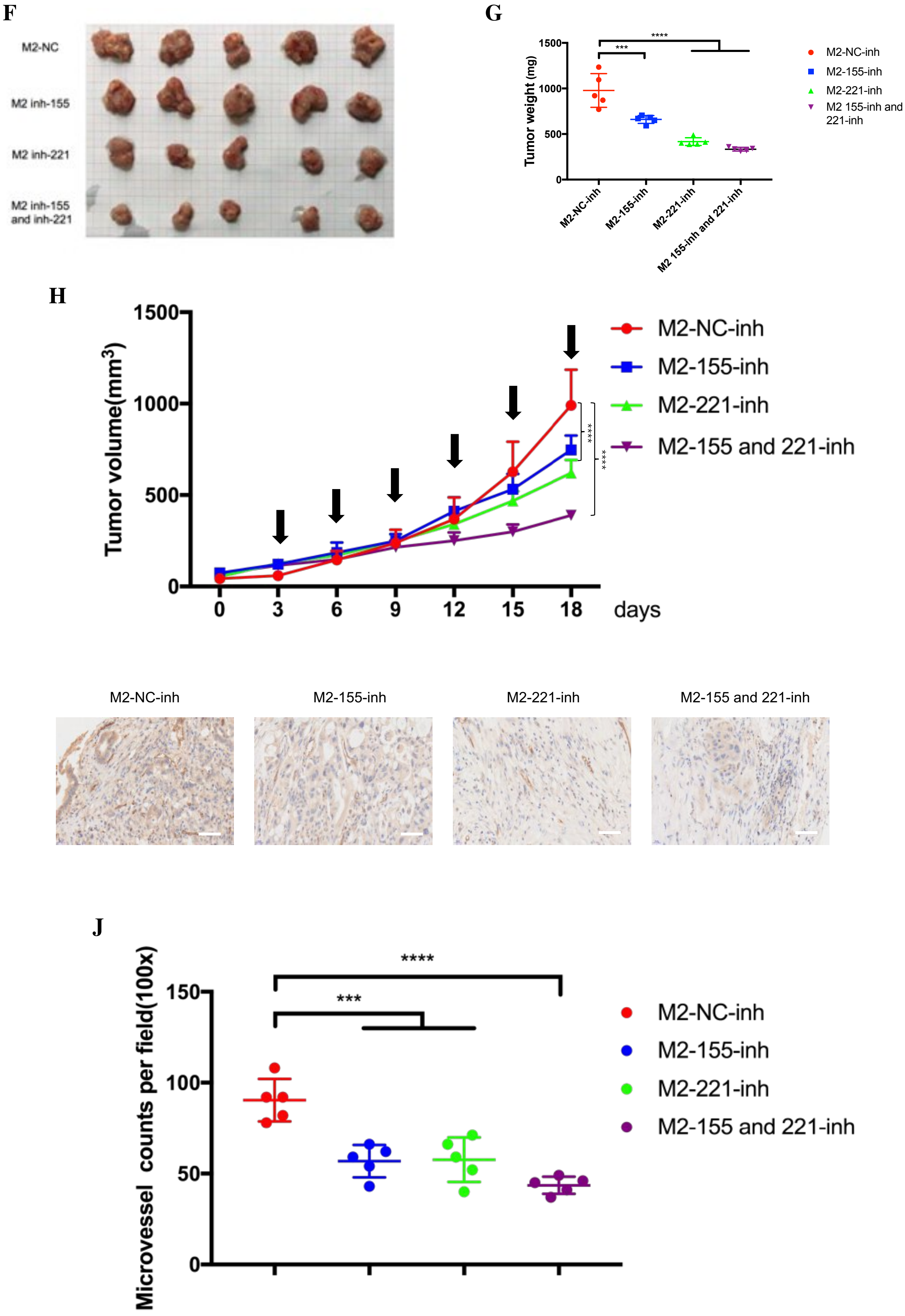
F
M2-NC
M2 inh-155
M2 inh-221
M2 inh-155 and inh-221
G
Tumor weight (mg)
1500
1000
500
0
****
***
M2-NC-inh
M2-155-inh
M2-221-inh
M2 155-inh and 221-inh
M2-NC-inh
M2-155-inh
M2-221-inh
M2 155-inh and 221-inh
H
Tumor volume(mm³)
1500
1000
500
0
0
3
6
9
12
15
18
days
****
****
M2-NC-inh
M2-155-inh
M2-221-inh
M2-155 and 221-inh
I
M2-NC-inh
M2-155-inh
M2-221-inh
M2-155 and 221-inh
J
Microvessel counts per field(100x)
150
100
50
0
****
***
M2-NC-inh
M2-155-inh
M2-221-inh
M2-155 and 221-inh

K

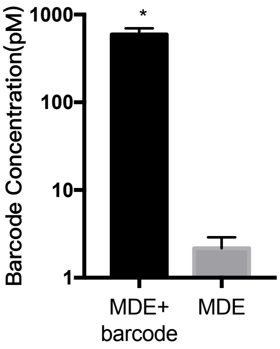

L

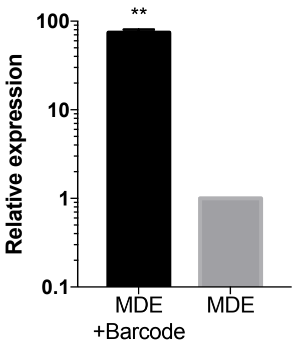

(a) Tumor image of each group.

(b) Data statistics of the tumors weight in each group.

(c) The tumor growth curve shows the tumor size measured every 3 days, and the arrow represents the injected exosomes.

(d) The representative IHC graph of tumor tissue indicates endothelial cells (by CD31) in each group. (scale bar 100μm).

(e) Data statistics of the MVD in each group of tumor tissue.

(f) Tumor image of each group.

(g) Data statistics of the tumors weight in each group.

(h) The tumor growth curve shows the tumor size measured every 3 days, and the arrow represents the injected exosomes.

(i) The representative IHC graph of tumor tissue indicates endothelial cells (by CD31) in each group. (scale bar 100μm).

(j) Data statistics of the MVD in each group of tumor tissue.

(k) M2 macrophages were transfected with synthetic 75nt dsDNA barcode or control. After 48 hours, exosomes were purified from CM. qPCR was done to assess barcode concentration inside MDE. Barcode concentration in barcode transfected MDE (MDE+Barcode) was 593pM, compared with 2.155pM in controls (MDE) $p<0.05$.

(l) qPCR for detection of the ds-DNA barcode, sorting from tumor tissue(CD31+).

Supplemental data 4: Top20 in RNA-seq of miRNA in macrophage-derived exosomes.

id	log2(fc)	Pvalue
mmu-miR-5112	12.3245181	4.82E-07
mmu-miR-155-5p	11.3245181	0.00097925
mmu-miR-320-5p	10.8099449	0.00782344
mmu-miR-381-3p	10.8099449	0.00782344
mmu-miR-382-5p	10.8099449	0.00782344
mmu-miR-18a-5p	10.5875525	0.01564141
mmu-miR-324-5p	10.5875525	0.01564141
mmu-miR-370-3p	10.5875525	0.01564141
mmu-miR-93-3p	10.5875525	0.01564141
mmu-miR-125b-2-3p	10.3245181	0.03127344
mmu-miR-221-5p	10.3245181	0.03127344
mmu-miR-872-5p	10.3245181	0.03127344
mmu-miR-3535	3.50802647	6.56E-59
mmu-miR-149-5p	3.34596164	0.00052118
mmu-miR-501-3p	2.93503471	2.04E-27
mmu-miR-409-5p	2.50946689	1.39E-16
mmu-miR-486a-5p	2.47376256	6.88E-85
mmu-miR-486b-5p	2.46865134	3.12E-82
mmu-miR-421-3p	2.30848694	0.0005377
mmu-miR-146b-5p	2.27779706	9.83E-51

Supplemental data 5: M2 macrophage derived exosomal miR-155-5p and miR-221-5p induce angiogenesis by targeting E2F2.

A

miR-155-5p

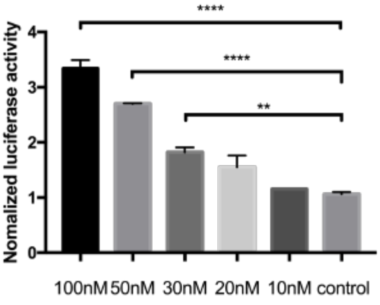

B

miR-221-5p

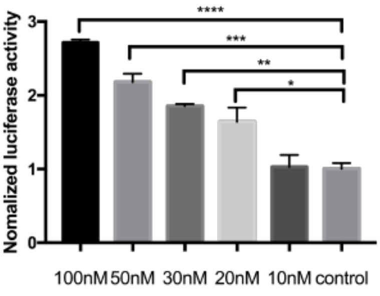

C

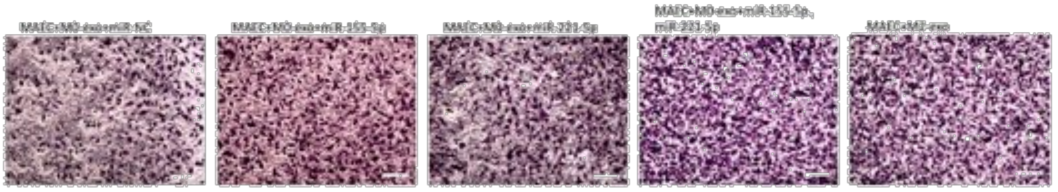

D

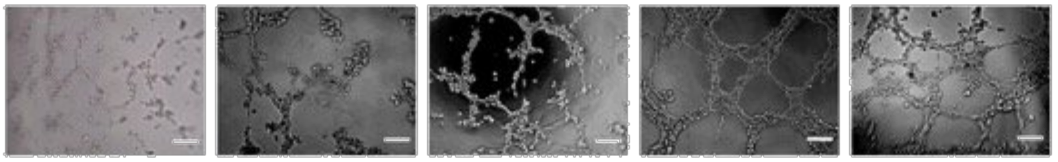

E

0h

24h

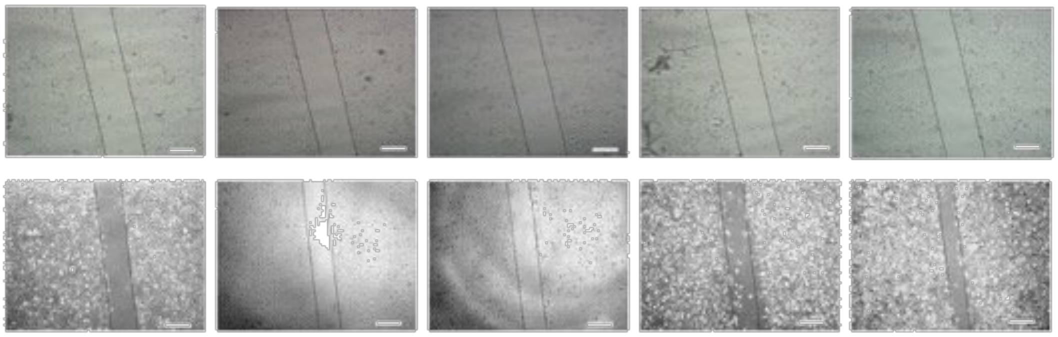

F

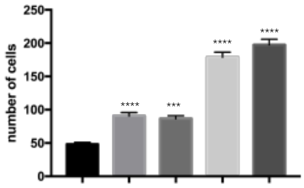

G

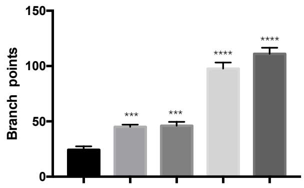

H

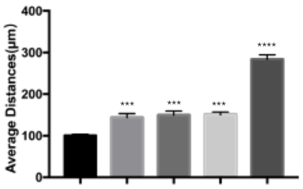

I

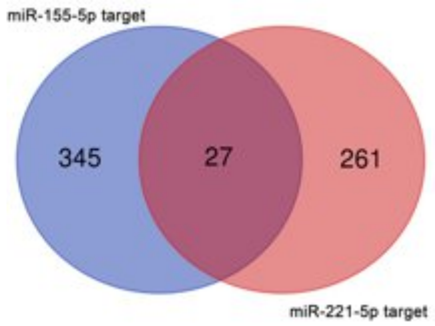

J

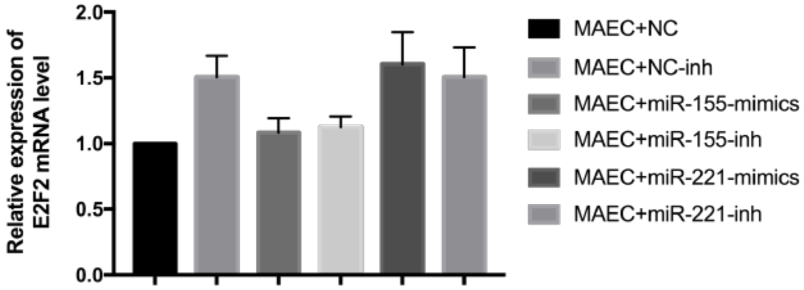

K

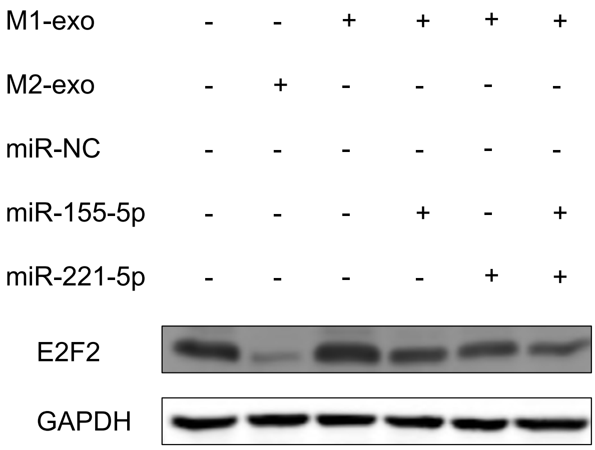

L

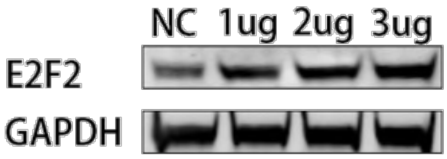

(a) plasmid containing wild-type E2F2 3'-UTR predicted miR-155-5p or/and miR-221-5p target sequences were transfected into BMDM, along with miR-155-5p mimics, and relative luciferase activity was detected.

(b) plasmid containing wild-type E2F2 3'-UTR predicted miR-155-5p or/and miR-221-5p target sequences were transfected into BMDM, along with miR-155-5p mimics, and relative luciferase activity was detected.

(c) Representative micrographs of the transwell assay (magnification 100×) . Number of cells were calculated per high-power field from three independent experiments(scale bar 200μm)..

(d) Representative micrographs of tube formation assay(magnification 200×).The number of branch points were calculated by imageJ (scale bar 100μm).

(e) Representative micrographs of the 24h average distance of wound-healing assay(scale bar 100μm).

(f-h) Data statistics of transwell, wound-healing and tube formation assays. ***$p<0.001$, ****$p<0.0001$

(i) The result showed that there were a total of 27 common targets for miR-155-5p and miR-221-5p.

(j) qPCR indicate the expression of E2F2 in MAEC transfected with miR-155-5p or miR-221-5p mimics/inhibitor.

(k) E2F2 level of MAEC after cocultured with M1-exosomes which were loaded miRNA-mimics.

(l) Transfection efficiency of E2F2 Overexpression plasmid detected by Western Blot.

Supplemental Methods

Real-time PCR

Total RNA from cells and exosomes was extracted using Trizol reagent (Invitrogen, Carlsbad, CA, USA) and complementary DNA was synthesized with Reverse Transcription system (Ribo, China) according to the manufacturer's instructions. For miR detection, qPCR was performed with commercial SYBR Green PCR Master Mix (Ribo, China) using 1 ng of small RNA per reaction. U6 was used as an internal control. In addition, for the miRNA in exosomes, cel-miR-39-5p was used as an External control and the relative expression levels were evaluated using the $^{\Delta\Delta}$Ct method. The primers were purchased from GenScrip(China) and their sequences used were shown in（supplement2）.

MiRNA, RNA interference and construction of adenoviral systems

Synthetic miRNAs, miRNAs inhibitors and Cy3 labeling miRNAs were synthesized and purified by RiBo (RiboBio Co., Guangzhou, China). RNA oligonucleotides were transfected by using lipofectamine 3000 (Invitrogen, Carlsbad, CA, USA) and media was replaced 6h after transfection. Cy3 labeled miRNAs were transfected into macrophages with using lipofectamine 3000 (Invitrogen, Carlsbad, CA, USA). Macrophages containing Cy3-miRNA were co-cultured with MAEC cells and samples were examined using fluorescence microscope.

DiI-labeled exosomes transfer assay

Purified exosomes isolated from M2 macrophages were suspended in 1000μl PBS and incubated with 10μl DiI for 30 min at 37°C.

Monoclonal antibody

Anti-CD206(ab8918), anti-CD163(ab182422), anti-CD86(ab213044), anti-CD-31(ab28364), anti-CD81(ab219209), anti-CD9(ab92726), anti-LAMP2(ab199946), anti-GAPDH(ab181602) and anti-E2F2(ab235837) antibodies were purchased from Abcam.

Bioavailability assay

We synthesized a unique 75nt long dsDNA "barcode fragment", which was transfected to Macrophages as previously described. Simply, 1μM of barcode DNA were transfected into Macrophages using Lipofectamine.

DNA Barcode:CCCTTGAACCTCCTCGTTCGACCAGCTACCTGAGTATCGTCCCTCGAACGCTACAGTAGCTAGCCTGTGGCAGAG,

forward primer: 5' CCC TTG AAC CTC CTC GTT CG,

reverse primer: 5' CTC TGC CAC AGG CTA GCT ACT.

To assess the copy number of barcode in exosomes purified from the transfected Macrophages, DNA was extracted from exosomes and qPCR was performed. The copy number was calculated using a barcode standard curve. Exosomes purified from non-transfected Macrophages were used as controls. After 48 hours, exosomes were purified from CM. qPCR was done to assess barcode concentration inside MDE. Two days after the last injection, mice were sacrificed and the tumors were separated and dissociated to a single cell suspension. Cells were stained using anti-wide spectrum cytokeratin antibody and anti-CD31 antibody (Abcam, Cambridge, UK), and sorted by FACS Aria II cell sorter (BD Biosciences, San Jose, CA) to cytokeratin negative, CD31 positive cells. DNA was purified and cleaned from excess fluorophores using Mutisource Genomic DNA Miniprep Kit (Axygen, USA). qPCR was performed for barcode and β-actin gene was use for normalization.
